# Associations between Activity Pacing, Fatigue, and Physical Activity in Adults with Multiple Sclerosis: A Cross Sectional Study

**DOI:** 10.3390/jfmk5020043

**Published:** 2020-06-15

**Authors:** Ulric S. Abonie, Femke Hoekstra, Bregje L. Seves, Lucas H. V. van der Woude, Rienk Dekker, Florentina J. Hettinga

**Affiliations:** 1 Department of Physiotherapy and Rehabilitation Sciences, University of Health and Allied Sciences, Ho PMB 31 Volta Region, Ghana; uabonie@uhas.edu.gh; 2 School of Sport, Rehabilitation and Exercise Science, University of Essex, Colchester CO4 3SQ, Essex, UK; 3 Center for Human Movement Sciences, University Medical Center Groningen, University of Groningen, PO Box 72, 9700 AB Groningen, The Netherlands; fhoekstr@mail.ubc.ca (F.H.); b.l.seves@umcg.nl (B.L.S.); l.h.v.van.der.woude@umcg.nl (L.H.V.v.d.W.); 4 Department of Rehabilitation, University Medical Center Groningen, University of Groningen, PO Box 72, 9700 AB Groningen, The Netherlands; r.dekker01@umcg.nl; 5 Department of Sport, Exercise and Rehabilitation, Northumbria University, Newcastle upon Tyne NE1 8SB, UK

**Keywords:** activity pacing, multiple sclerosis, perceived risk of overactivity, perceived fatigue, health-related quality of life, rehabilitation

## Abstract

Fatigue is common in people with multiple sclerosis (MS). Activity pacing is a behavioral way to cope with fatigue and limited energy resources. However, little is known about how people with MS naturally pace activities to manage their fatigue and optimize daily activities. This study explored how activity pacing relates to fatigue and physical activity in people with MS. Participants were 80 individuals (60 females, 20 males) with a diagnosis of MS. The participants filled in questionnaires on their activity pacing, fatigue, physical activity, and health-related quality of life, 3–6 weeks before discharge from rehabilitation. The relationships between the variables were examined using hierarchical regression. After controlling for demographics, health-related quality of life, and perceived risk of overactivity, no associations were found between activity pacing and fatigue (β = 0.20; t = 1.43, *p* = 0.16) or between activity pacing and physical activity (β = −0.24; t = −1.61, *p* = 0.12). The lack of significant associations between activity pacing and fatigue or physical activity suggests that without interventions, there appears to be no clear strategy amongst people with MS to manage fatigue and improve physical activity. People with MS may benefit from interventions to manage fatigue and optimize engagement in physical activity.

## 1. Introduction

Symptoms of fatigue are among the most frequently reported and strongest predictors of functional disability in people with multiple sclerosis (MS) [1,2,3]. The experience of fatigue and perceived fatigability (changes in the sensations that regulates effort and endurance) draws behavioral adaptations, such as limiting the engagement in activities resulting in underactivity, or a lifestyle characterized by periods of overactivity followed by long extensive rest periods [4,5,6,7]. However, both underactivity and overactivity are linked with disability [8].

Despite growing efforts to manage fatigue through exercise interventions in people with MS, studies investigating the effect of exercise interventions report a high number of dropouts, and identified that participants struggle to continue engaging in physical activity post-intervention [9,10]. This warrants the need to explore ways to enable long-term adoption of a physically active lifestyle.

Activity pacing is a self-management strategy that can help alter often-occurring inefficient activity patterns (underactivity and overactivity) and stimulate long-term engagement in an active lifestyle [11]. It involves dividing one’s daily activities into smaller, manageable pieces to manage fatigue, and to maintain a steady activity pace, whilst reducing relapses [12,13]. However, current literature on how people naturally pace activities in daily life is limited and inconclusive [11,12,13,14,15,16]; some studies show that activity pacing is associated with higher levels of fatigue and lower physical activity [16,17], while others show the opposite or no association [8,18], and no clear strategies are available in rehabilitation treatment to optimize activity pacing to improve engagement in physical activity [14]. Similarly, quality of life has been proposed to impact activity pacing [8].

It is notable that most of the above studies aimed to explore issues in a range of chronic disabling conditions and did not focus on MS specifically. Thus while findings from these studies [8,13,14,15,16,17,18] contribute to our understanding of activity pacing, their broader focus with regards to multiple health behaviors and mixed populations may have resulted in failure to elicit key issues specific to engagement in physical activity for people with MS. Currently no study has explored people with MS in a natural approach to activity pacing, and its relations to fatigue and physical activity.

Understanding these associations can help guide and tailor rehabilitation treatment efforts for people with MS and promote an active lifestyle in this population. The aim of this study was to examine reported engagement in pacing and how it relates to fatigue and physical activity in people with MS just before discharge from rehabilitation, controlling for demographics, health-related quality of life, and perceived risk of overactivity. Based on the expectation that activity pacing would be an adaptive strategy to manage fatigue and optimize daily activities [14,17], we hypothesized that reported engagement in pacing would be associated with a decrease in fatigue and an increase in physical activity.

## 2. Materials and Methods

### 2.1. Design

This study was part of a multicenter longitudinal study (Rehabilitation, Sports, and Active lifestyle; ReSpAct) to evaluate the nationwide implementation of an active lifestyle program (Rehabilitation, Sports, and Exercise; RSE) among people with a wide range of chronic diseases and/or physical disabilities in Dutch rehabilitation [19,20]. Participants received either inpatient or outpatient rehabilitation at rehabilitation centers and departments of rehabilitation in hospitals because of MS. The current study uses a cross-sectional design based on baseline measurement (3–6 weeks before discharge from rehabilitation) of activity pacing behaviors, fatigue, physical activity, and health-related quality of life of people with MS, selected from the ReSpAct dataset. The study procedures were approved by the ethics committee of the Center for Human Movement Sciences of the University Medical Center Groningen, University of Groningen (reference: ECB/2013.02.28_1) and at participating institutions.

### 2.2. Participants

Participants were recruited upon referral to the participating rehabilitation institutions across the Netherlands. Potential participants received information on study rationale and procedures, had questions answered, and were checked for the inclusion criteria. Participants were included in this study if they were 18 years and older, had a diagnosis of MS, had received rehabilitation care or treatment based on medicine consultation within one of the participating rehabilitation institutions, and participated in the ‘RSE’ program. Participants were excluded from the study if they were not able to complete the questionnaires, even with help, or participated in another physical activity stimulation program. Eligible participants who volunteered signed an informed consent form.

### 2.3. Procedure

Enrolled participants were assessed through a standardized baseline measurement, which consisted of filling out a set of questionnaires on paper or digitally [19,21,22,23]. As part of the full questionnaire and producer, first, participants indicated which physical activities they perform in the context of the rehabilitation treatment and on their own initiative by filling out an adapted version of the short questionnaire to assess health enhancing physical activity (SQUASH) [21]. Secondly, participants filled out short questionnaires on their perceived engagement in pacing, risk of overactivity, and fatigue [19,22]. Lastly, participants filled out a questionnaire on their health-related quality of life [23].

### 2.4. Measures

#### 2.4.1. Primary Measures

Fatigue severity was measured using the Fatigue Severity Scale (FSS) [22], a valid and reliable questionnaire to determine the impact of fatigue in people with MS [24]. The participants scored the nine items of the questionnaire on a scale of 1–7 (1, completely disagree; 7 completely agree). Mean fatigue score based on an average of the nine items was used. The mean fatigue score ranges from 1 to 7. A mean FSS score ≥4 was adopted as the cut-off for clinically significant fatigue [25].

Physical activity was assessed using an adapted version of the Short Questionnaire to Assess Health-Enhancing Physical Activity [21]. The questionnaire is a self-reported recall measure to assess daily physical activity based on an average week in the past month. The original questionnaire has demonstrated good test–retest reliability and internal consistency and moderate concurrent validity in ordering participants according to their level of physical activity [21,26,27]. Some minor changes were made to make the SQUASH applicable for people with a chronic disease or physical disability. Specifically, within the domains ‘commuting activities’, ‘leisure-time’, and ‘sports activities’, the items ‘wheelchair riding’ and ‘hand cycling’ were added. Also, ‘tennis’ was modified as ‘(wheelchair) tennis’. Total minutes of physical activity per week was calculated by multiplying frequency (days/week) and duration (minutes/day) for each activity.

Reported engagement in pacing was assessed with the ‘engagement in pacing’ subscale of the Activity Pacing and Risk of Overactivity Questionnaire [19]. This questionnaire was developed for use in the ReSpAct study [19]. The engagement in pacing subscale reflected reported engagement in pacing within daily routines and was the primary outcome in the current study. Participants scored the five items of the subscale on a scale of 1–5 (1, never; 2, rarely; 3, sometimes; 4, often; 5, very often). The mean subscale score ranged from 1 to 5, with higher score indicating high engagement in pacing.

Appendix A shows the preliminary validation metrics of the questionnaire. In summary, the sampling adequacy tested with the Kaiser–Myer–Olkin (KMO) and the Bartlett’s test of sphericity showed that the questionnaire had a KMO of 0.722, and Bartlett’s test was significant (*p* < 0.05), supporting a principal component analysis (PCA). Results of the PCA showed that there were two factors with an Eigen value >1.00, therefore based on the Kaiser’s criterion two components were chosen. Factor loadings were used to assign the items to the two components. The two components explained 60.50% of the total variance and there was a negative correlation between the two components of −0.115.

#### 2.4.2. Background Measures and Confounders

Background demographics included age, gender, and body mass index, which was calculated from self-reported body mass and height (body mass (kg)/height^2^ (m^2^)).

To assess health-related quality of life, the RAND-12-Item Health Survey (RAND-12) [23] was used. RAND-12 assesses seven health domains; general health, physical functioning, role limitations due to physical health problem bodily pain, role limitations due to emotional problems, vitality/mental health, and social functioning. The RAND-12 was scored using the recommended scoring algorithm for calculating general health [28], a composite score of person’s health-related quality of life. Scores ranged from 18 to 62. A high score indicated better health-related quality of life. The RAND-12 has been proven to be a valid and reliable measure of health-related quality of life [29].

The ‘risk of overactivity’ subscale of the Activity Pacing and Risk of Overactivity Questionnaire [19] was used to measure perceived risk of overactivity within daily routines. Participants scored the two items of the subscale on a scale of 1–5 (1, never; 2, rarely; 3, sometimes; 4, often; 5, very often). The mean score ranged from 1 to 5, with higher score indicating high perceived risk of overactivity.

### 2.5. Data Analysis

Data were analyzed using IBM Statistical Package for the Social Sciences version 23.0 [30]. Based on descriptive statistics and visual inspection of frequency distributions, data were normally distributed. All values were reported using descriptive statistics of means, standard deviations, and interquartile ranges to summarize characteristics of participants. To ensure there was no multicollinearity, bivariate Pearson correlations were conducted to examine basic between-person associations among demographic and study variables, prior to testing the study hypotheses (variables were not highly correlated with each other, *r* < 0.8).

Hierarchical linear regression was used to test the study hypotheses. This statistical approach was optimal for adjustment for confounders, as we wanted to determine whether there were relationships between engagement in pacing and fatigue, and between engagement in pacing and physical activity after controlling for demographics, health-related quality of life, and perceived risk of overactivity.

To examine how engagement in pacing was related to fatigue and physical activity, two hierarchical regression analyses were conducted with fatigue or physical activity as the dependent variable, and engagement in pacing as the independent variable. Age, gender, body mass index, health-related quality of life, and perceived risk of overactivity were confounders.

These demographics and confounders were included in the models based on the fact that they are general demographic variables of interest in studies on physical activity behaviour and fatigue experience, and on known associations with perceived fatigability and physical activity behaviour [18,31]. We chose to analyze our data using these models based on the literature and our expectation that activity pacing may be a positive strategy to manage fatigue and optimize daily activities [14,15].

In both models, at the first step, gender, age, and body mass index were entered. At the second step, health-related quality of life and perceived risk of overactivity were entered, and at the third step engagement in pacing was entered. In both models, the variance inflation factors (VIFs) were examined for multicollinearity.

## 3. Results

Of the 89 participants included in the study, nine participants had incomplete data and were therefore excluded from the analysis. Characteristics of the sample (*N* = 80) are shown in Table 1. Of the sample, 75% were female *(n* = 60) and the mean age was 44 ± 11 years. The majority of the sample *(n* = 73, 91.3%) were scored as having clinically significant fatigue on the FSS (FSS score > 4). We found that 85.61% (*n* = 69) of the participants lived independently and 33.6% (*n* = 69) had a university education. The sample was, on average, overweight according the World Health Organization standards (body mass index ≥ 25.0 kg/m^2^).

Bivariate Pearson correlations (Table 2) showed that the variables were not strongly correlated with each other, providing support for the decision to include them into the primary analyses. Fatigue and health-related quality of life had the highest modest correlation (*r* = −0.41). The next modest correlations were between engagement in pacing and health-related quality of life (*r* = −0.27), and between engagement in pacing and fatigue (*r* = 0.27). These were followed by the correlations between engagement in pacing and physical activity (*r* = −0.25), and between engagement in pacing and age (*r* = 0.24). All other bivariate correlations were of modest magnitude (*r* ≤ ±0.22).

### 3.1. Primary Analyses

#### 3.1.1. Relationship between Engagement in Pacing and Fatigue

Results of the relationship between engagement in pacing and fatigue, controlling for demographics and confounders (Table 3), showed no association between engagement in pacing and fatigue (β = 0.198; *t* = 1.43, *p* = 0.16). Among the confounders, health-related quality of life was negatively related to fatigue (β = −0.341; *t* = −2.57, *p* = 0.03).

#### 3.1.2. Relationship between Engagement in Pacing and Physical Activity

Results of the relationship between engagement in pacing and physical activity, controlling for demographics and confounders (Table 4), revealed no association between engagement in pacing and physical activity (β = −0.242; *t* = −1.61, *p* = 0.12). None of the demographics and confounders was related to physical activity (*p* ≥ 0.05).

For all analyses, the VIFs were low showing that there was no problem of multicollinearity (range: 1.04–1.30).

## 4. Discussion

This study explored relations of reported engagement in pacing with fatigue and physical activity, while controlling for demographics, health-related quality of life, and perceived risk of overactivity in adults with MS and found no associations between engagement in pacing and fatigue or and physical activity. These findings were similar to the findings of Murphy et al. [18] but did not support our hypothesis that engagement in pacing would be associated with low fatigue and high physical activity. Regarding the confounders, health-related quality of life was negatively related to fatigue. Descriptive statistics showed people with MS demonstrated clinically significant fatigue complaints, which was similar to studies evaluating fatigue in the MS population [32], high engagement in pacing and a high perceived risk of preventing overactivity. The total minutes of physical activity level reported by participants in our study is consistent with previous research involving people with MS [6,33]. The FSS score (5.43 ± 1.11) and percentage of participants reporting clinically significant fatigue (91.3%) in our study were comparable with those reported in other studies involving people with MS [1,34,35]. In their studies, Weiland et al. [34] and Hadgkiss et al. [35] reported median FSS score of 4.9 (IQR 3.2–6.1) with 65.6% of the sample reporting clinically significant fatigue. Similarly, Merkelbach et al. [1] reported a mean FSS score of 4.4 ± 1.6, with 58.75% of the sample reporting clinically significant fatigue.

Bivariate correlation analysis conducted prior to the primary analyses revealed a moderate negative association between fatigue and health-related quality of life, indicating high fatigue was associated with low health-related quality of life. Furthermore, there was a weak negative association between engagement in pacing and health-related quality of life, suggesting that high engagement in pacing was associated with low health-related quality life. Together, these findings suggest that without interventions, there appears to be no clear strategy for using physical activity to ameliorate fatigue symptoms and improve quality of life amongst people with MS. This underscores the need to explore the potential of guiding and advising people with MS regarding optimal pacing behaviour and to develop therapeutic interventions.

A possible explanation for the lack of associations between reported engagement in pacing and fatigue or physical activity after controlling for demographic and confounding variables, coupled with the clinically significant fatigue found in this study, may be multiplicity in persons’ attitudes towards physical activity in relation to fatigue symptoms. People with MS who experience more disruption through fatigue in daily life may be consciously limiting their activities to prevent fatigue worsening, or exhibiting all-or-nothing behaviour, which is a lifestyle characterized by periods of overactivity (when feeling good) and as a consequence of that, feeling overtly fatigued afterwards, followed by long extensive rest periods to recover from residual symptoms or prevent symptoms re-occurring. For those consciously limiting their activities to prevent fatigue worsening, more engagement in pacing will most likely result in less physical activity, while for those exhibiting a lifestyle characterized by periods of overactivity and prolonged inactivity, more active engagement in pacing will most likely result in more physical activity, and thus when both attitudes are present in the subject population no relations between activity pacing and physical activity may be found.

This further highlights the importance to explore the natural use of activity pacing in relation to what we know from literature to help guide treatment efforts for people with MS. Tailored advice and goal-directed interventions on how to approach activity effectively, such as guidance on optimal use of pacing, might be beneficial for people with MS. For example, people who avoid physical activity in anticipation to fatigue might score high on engagement in pacing but may need advice to engage more in physical activity, and could be provided with a graded consistent program of physical activity to increase their health, as well as be given information and strategies to help change their beliefs that “I should do less if I am tired” or “symptoms are always a sign that I am damaging myself.” Similarly, people who have developed an all-or-nothing behaviour style might need advice to be more aware of anticipatory ways of engaging in pacing to develop a consistent pattern of paced activity and rest.

To our knowledge this is the first study to tap into the experiences of people with MS during their daily routines and explore the associations between engagement in pacing, fatigue, and physical activity. Adequate management of fatigue might be essential to improve health and wellbeing in people with MS, based on the findings of this study and previous literature that revealed most people with MS experience high levels of fatigue throughout the day [31]. Though the sample size in this study was substantial for this population (*N* = 80), it would be useful to replicate these analyses in a larger sample to obtain more precise estimates of the model parameters while controlling for confounders. Furthermore, the adapted SQUASH, and the Activity Pacing and Risk of Overactivity Questionnaire used in this study are recent and have undergone limited validity and sensitivity testing, which may have influenced the study findings. Currently, further studies on the validity of these measurements and usage for the current purposes are being conducted. Although self-report measures are more feasible in population studies, they are susceptible to biases as they involve recalling activities (over days, weeks, or months) that could lead to underreporting or overreporting. Using an objective device would allow to examine more macro levels of activity and is warranted in future study.

To optimize generalizability within the population of people living with MS, this study was conducted solely in people with MS. Generalizability to other populations might therefore be limited, as findings may vary per condition [14]. Unfortunately, there was a lack of information on participants’ MS type and MS disability in this study, which limits the ability to draw firm conclusions. These variables could influence the study findings. Lastly, the weak bivariate correlations between reported engagement in pacing and fatigue, and between reported engagement in pacing and physical activity found may have accounted for the lack of associations after controlling for demographics, health-related quality of life, and perceived risk of overactivity. It is worth noting that although participants received rehabilitation treatment as part of the larger multicenter study, a structured activity pacing program was not included and we do not think this has influenced the findings of this study. Future studies should further explore how engagement in pacing and perceived risk of overactivity relate to performance of activities of daily living, to allow for firm conclusions and help advice people with MS on how to engage in an active lifestyle. Additionally, exploratory studies on how activity pacing behaviour might affect physical activity, fatigue, and health-related quality of life over a longer period of time are warranted. Such studies should explore higher versus lower fatigue group in terms of clinical fatigue cut-off point (FSS > 4) or a median split, to help better understand associations.

## 5. Conclusions

This study examined the relationships between reported engagement in pacing and fatigue and physical activity in people with MS, while controlling for demographics, perceived risk of overactivity, and health-related quality of life. No associations were found between reported engagement in pacing and fatigue, or between reported engagement in pacing and physical activity. We found that low health-related quality of life was associated with high fatigue. People with MS might benefit from targeted interventions to better manage their fatigue and improve their health and wellbeing. Ascertaining engagement in pacing may be important to help tailor advice on optimal pacing behaviour for people with MS. There is a need to explore the potential of guiding and advising people with MS on activity pacing and develop therapeutic interventions.

## Figures and Tables

**Table 1:** Demographics of Participants (*N* = 80).

Variable	Mean ± SD or *N* (%)	Interquartile Range *	Scale Range
Age (years)	44.48 ± 10.67	38.00–52.00	22–68
Body mass index (kg/m^2^)	27.28 ± 6.91	23.04–32.41	18–53
Number of women (%)	60 (75)		
Living situation (lives alone)	69 (85.61)		
High education level ^a^	27 (33.6)		
Engagement in pacing	3.74 ± 0.71	3.30–4.20	1–5
Perceived risk of overactivity	3.73 ± 0.86	3.00–4.50	1–5
Fatigue severity	5.43 ± 1.11	4.78–6.17	1–7
Physical activity (minutes/week)	1585.64 ± 1103.51	780.00–2070.00	
Health-related quality of life	34.35 ± 7.73	28.03–40.26	18–62

* Interquartile range of the 25th percentile and the 75th percentile. ^a^ Completed university or higher.

**Table 2:** Bivariate Pearson correlations of all variables in the hierarchical linear regression models.

Variables	2	3	4	5	6	7	8
1. Engagement in pacing	−0.21	0.27 *	−0.25 *	−0.27 *	0.24 *	0.05	−0.20
2. Perceived risk of overactivity		0.19	0.14	−0.16	−0.10	−0.04	0.21
3. Fatigue			−0.14	−0.41 **	0.04	0.04	−0.02
4. Physical activity				−0.02	−0.15	0.08	0.09
5. Health-related quality of life					0.12	−0.05	−0.4
6. Age						−0.22	−0.17
7. Gender							−0.12
8. Body mass index							1

* Correlation is significant at the 0.05 level. ** Correlation is significant at the 0.01 level.

**Table 3:** Hierarchical linear regression model showing the relationship between engagement in pacing and fatigue.

Variable	β	B	SE	df	t	*p*-Value
Age	0.058	0.006	0.014	3, 55	0.436	0.664
Gender	0.065	0.155	0.302	3, 55	0.515	0.609
Body mass index	−0.011	−0.002	0.021	3, 55	−0.086	0.931
Health-related quality of life	−0.341	−0.049	0.019	2, 53	−2.568	0.013
Perceived risk of overactivity	0.188	0.243	0.167	2, 53	1.454	0.152
Engagement in pacing	0.198	0.307	0.215	1, 52	1.431	0.158

β, Standardized regression coefficients from the complete regression model accounting for all variables; B, unstandardized regression coefficients from the complete regression model accounting for all variables; df, degree of freedom; SE, standard error of B. Note: In this model, fatigue was the dependent variable, engagement in pacing was an independent variable, and the other variables were confounders.

**Table 4:** Hierarchical linear regression model showing the relationship between engagement in pacing and physical activity.

Variable	β	B	SE	df	t	*p*-Value
Age	−0.054	−5.555	15.028	3, 55	−0.370	0.713
Gender	0.084	198.685	327.197	3, 55	0.607	0.546
Body mass index	0.029	4.558	22.309	3, 55	0.204	0.839
Health-related quality of life	−0.069	−9.8272	0.678	2, 53	−0.475	0.637
Perceived risk of overactivity	0.067	86.563	181.272	2, 53	0.478	0.635
Engagement in pacing	−0.242	−3733.690	232.825	1, 52	−1.605	0.115

β, Standardized regression coefficients from the complete regression model accounting for all variables; B, unstandardized regression coefficients from the complete regression model accounting for all variables; df, degree of freedom; SE, standard error of B. Note: In this model, physical activity was the dependent variable, engagement in pacing was an independent variable, and the other variables were confounders.

## Appendix A

**Table A1:** Factor loadings of the seven items of the Activity Pacing and Risk of Overactivity Questionnaire using Principal Component Analysis with oblique rotation.

Items	Factor 1	Factor 2
A. During the day I plan several moments to recover.	0.73 *	0.04
B. I perform my activities at a slow pace.	0.65 *	−0.13
C. When performing my activities, I take my fatigue into account.	0.79 *	0.00
D. When I’m engaged in an activity, I find it difficult to stop timely.	0.05	0.88 *
E. I alternate intensive activities with less intensive activities.	0.70 *	0.08
F. I divide my activities over the day.	0.74 *	−0.05
H. I find it hard to limit my activities.	−0.06	0.87

Factor 1: Engagement in pacing; Factor 2: Perceived risk of overactivity. * Loadings that can be explicitly assigned to a single factor (factor loading >0.40).
